# Correction: A highly sensitive and selective fluorescent probe for quantitative detection of Al^3+^ in food, water, and living cells

**DOI:** 10.1039/c9ra90037c

**Published:** 2019-05-30

**Authors:** Qian Jiang, Mingxin Li, Jie Song, Yiqin Yang, Xu Xu, Haijun Xu, Shifa Wang

**Affiliations:** College of Chemical Engineering, Nanjing Forestry University Nanjing 210037 P. R. China; Department of Chemistry and Biochemistry, University of Michigan-Flint Flint MI 48502 USA; College of Light Industry and Food, Nanjing Forestry University Nanjing 210037 P. R. China; Co-Innovation Center of Efficient Processing and Utilization of Forest Resources, Nanjing Forestry University Nanjing 210037 P. R. China

## Abstract

Correction for ‘A highly sensitive and selective fluorescent probe for quantitative detection of Al^3+^ in food, water, and living cells’ by Qian Jiang *et al.*, *RSC Adv.*, 2019, **9**, 10414–10419.

The authors regret that the details of the affiliations were incorrectly shown in the original manuscript. The corrected list of affiliations is as shown herein.

The Royal Society of Chemistry apologises for these errors and any consequent inconvenience to authors and readers.

## Supplementary Material

